# *In silico* analysis of mismatches in RT-qPCR assays of 177 SARS-CoV-2 sequences from Brazil

**DOI:** 10.1590/0037-8682-0657-2020

**Published:** 2020-11-25

**Authors:** Renan da Silva Santos, Raissa Souza Caminha Bret, Ana Cristina de Oliveira Monteiro Moreira, Adriana Rolim Campos, Angelo Roncalli Alves e Silva, Danielle Malta Lima, Kaio Cesar Simiano Tavares

**Affiliations:** 1 Universidade de Fortaleza, Núcleo de Biologia Experimental (NUBEX), Fortaleza, CE, Brasil

**Keywords:** SARS-CoV-2, COVID-19, Polymerase chain reaction, Diagnosis, Mismatches

## Abstract

**INTRODUCTION:**

Quantitative reverse transcription polymerase chain reaction (RT-qPCR) can detect the severe acute respiratory syndrome Coronavirus-2 (SARS-CoV-2) in a highly specific manner. However, a decrease in the specificity of PCR assays for their targets may lead to false negative results.

**METHODS:**

Here, 177 high-coverage complete SARS-CoV-2 genome sequences from 13 Brazilian states were aligned with 15 WHO recommended PCR assays.

**RESULTS:**

Only 3 of the 15 completely aligned to all Brazilian sequences. Ten assays had mismatches in up to 3 sequences and two in many sequences.

**CONCLUSION:**

These results should be taken into consideration when using PCR-based diagnostics in Brazil.

Coronavirus disease 2019 (COVID-19) is a severe acute respiratory syndrome (SARS) caused by the new SARS Coronavirus-2 (SARS-CoV-2, previously known as 2019-nCoV)^1^. COVID-19 exhibits a wide range of symptoms, such as fever, cough, shortness of breath or difficulty breathing, repeated shaking with chills, muscle pain, headache, sore throat, and new loss of taste or smell^2^. While some patients may not develop all of the symptoms, others might experience symptoms not mentioned in the previous list^2^. The outbreak started in Wuhan, China, in December 2019, followed by a rapid and massive worldwide spread, which led to the current pandemic^1^. The pandemic reached Brazil in March 2020; nevertheless, it has caused 2,442,375 confirmed cases and 87,618 confirmed deaths as of July 27, 2020^3^.

Several assays can be used to diagnose a patient with COVID-19. The polymerase chain reaction (PCR) technique is a molecular assay capable of detecting SARS-CoV-2 viral RNA with high specificity during the acute phase of infection. A wide variety of primers and probes were developed to detect this virus, mainly targeting the following genomic regions: ORF1ab, envelope genes (E), RNA-dependent RNA polymerase (RdRP), spike protein (S), and nucleocapsid (N)^4^. The high specificity of these molecular assays is directly related to the annealing specificity of the primer/probe to the genomic region. The World Health Organization (WHO) has recommended 26 primers and probes to be used in this type of diagnosis^5^.

Recent studies have shown that the rate and pattern of mutations in the SARS-CoV-2 genome differ depending on environmental conditions^6^. The present study investigated the specificity of fifteen primer and probe sets recommended by the WHO in 177 SARS-CoV-2 Brazilian genomes.

The SARS-CoV-2 genome sequences were obtained from the Global Initiative on Sharing Avian Influenza Data-EpiCoV (GISAID-EpiCoV) platform (https://www.gisaid.org/)^7^, an initiative for sharing genetic data of the SARS-CoV-2 virus. Only sequences submitted up to July 27, 2020, and complete genomes (above 29,000 bp) were included. A high coverage filter was applied, which means that only entries with less than 1% of undefined bases (stretches of NNNs) were tolerated, and Brazil was used as the location. The selected sequences were downloaded in FASTA format (Supplementary Data S1) and aligned using the ClustalW Multiple Alignment^8^ tool of the BioEdit biological sequence editor 7.2.6^21^. The genome used as reference was the first strain identified in Wuhan (China) (GenBank: NC_045512.2).

Nucleotide sequences with incomplete specificity of bases and constructions for nested PCR were not included, therefore some constructions were not taken into account in the analysis. The fifteen primers and probes constructions used here (sequences depicted at Figure 1) are recommended for the diagnosis of COVID-19 through RT-qPCR by the World Health Organization (WHO) and were originally available and published by independent institutions in seven countries^1^ ^,^ ^5^: China, France, USA, Japan, Germany, Hong Kong, and Thailand. All primers and probes were searched against the aligned genomes. 

A total of 177 SARS-CoV-2 complete genome sequences from Brazil, deposited before July 27, 2020, were obtained from GISAID-EpiCoV, and 470 strings were discarded as they did not achieve the requirement for high sequencing coverage. As shown in Figure 1, there are primers and probes with 100% hybridization against all sequences. However, many show with matching errors. For the N gene (Figure 1A), the Japanese PCR assay, NIID_2019-nCOV_N, showed 100% identity with the aligned sequences. However, most of the diagnostic assays recommended by WHO presented mismatches with the Brazilian SARS-CoV-2 sequences. The German Corman-N, Japanese N_Sarbeco, North American 2019-nCoV_N (N1, N2, and N3), and Thai NIH-TH_N presented 1 to 3 base pairs (bp) mismatches. The Chinese assays CN-CDC-N and HKU-N had a high frequency of mismatched sequences, 151 and 101, respectively, in view of the high prevalence of G28881A, G28882A, G28883C, and T29148C mutations. Unlike the mismatches found for the N gene, the targets against ORF1ab (Figure 1B) and E (Figure 1C) showed less frequent variability. The French nCoV_IP4 and Chinese CN-CDC-E assays demonstrated total identity to their motives. The other assays, nCoV_IP2, CN-CDC-ORF1ab, Charité-E, and E_Sarbeco showed low frequency of errors, such as 1 to 2 bp mismatches. 

**FIGURE 1:**
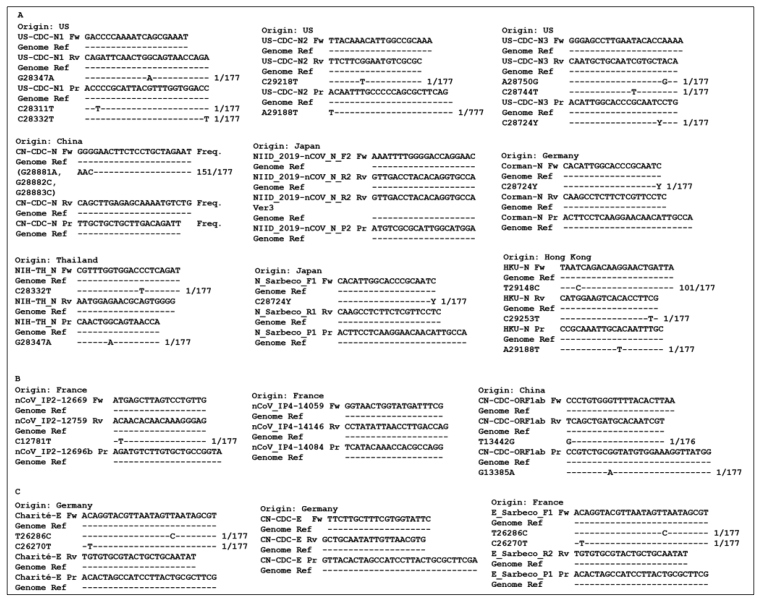
Alignment of PCR assays against the reference genome sequence (Genome Ref) and the mutated motifs. The occurrence of mismatch is indicated for each primer, forward (Fw) and reverse (Rv), and probe (Pr) for targets N (A), ORF1ab (B), and E (C). The frequency of a mutation among the 177 aligned sequences is shown on the side, if present.

Thirteen out of the 26 Brazilian states contributed with the deposit of sequences until the collection date. Most of these sequences were from two states, Rio de Janeiro (RJ) 49.71% (88/177) and São Paulo (SP) 27.69% (49/177) (Figure 2A). Three of the four most frequent mutations found, G28881A, G28882A, and G28883C, were observed mainly in sequences from RJ (54.30 %) and SP (26.49 %) (Figure 2B). However, interestingly, the T29148C mutation was highly common among sequences collected from RJ (74.25 %) and was rarely seen in the sequences collected in SP (3.96 %) (Figure 2C). The remaining eleven states had less participation in the number of genomes deposited so far. 

**FIGURE 2:**
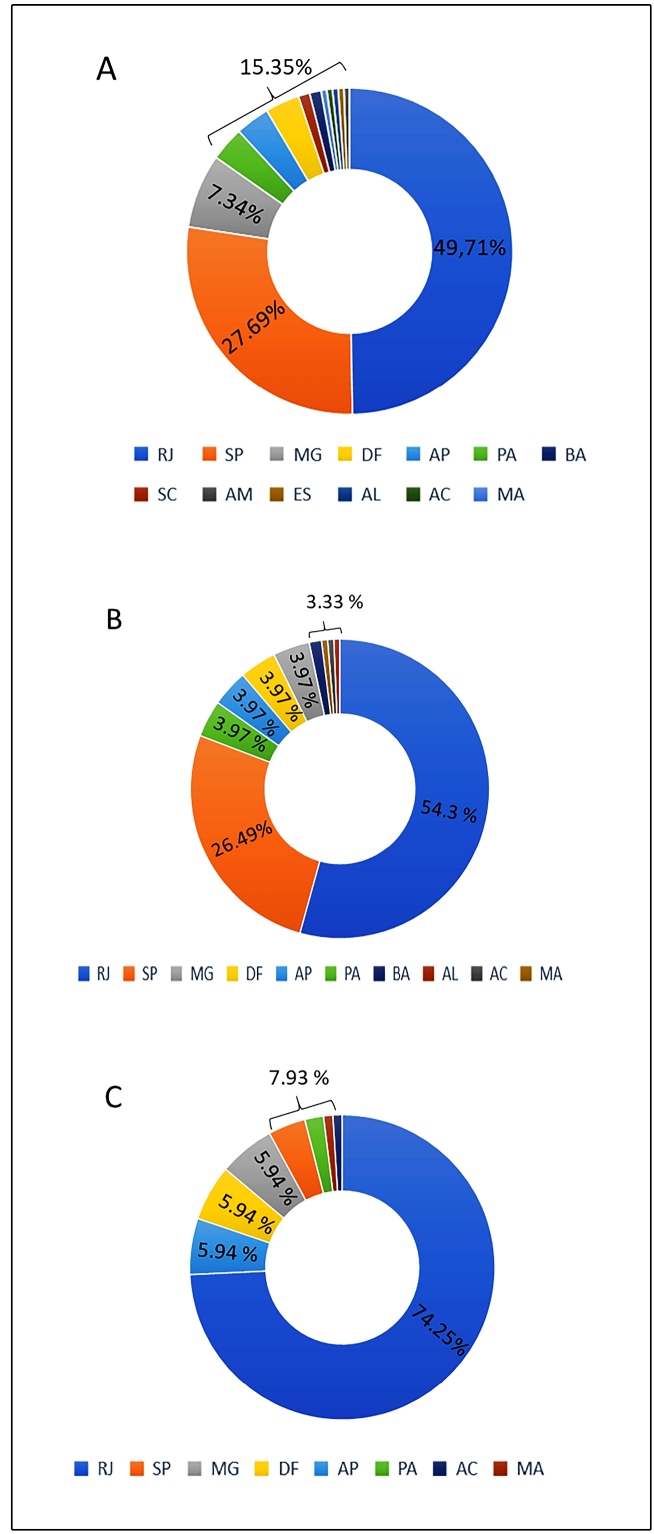
(A) Brazilian SARS-Cov-2 genome sequences deposited in the GISAID-EpiCoV platform, divided by state: Rio de Janeiro (RJ), São Paulo (SP), Minas Gerais (MG), Distrito Federal (DF), Amapá (AP), Pará (PA), Bahia (BA), Santa Catarina (SC), Amazona (AM), Espírito Santo (ES), Alagoas (AL), Acre (AC) and Maranhão (MA). Occurrence of the G28881A, G28882A, G28883C (B), and T29148C (C) mutations in each state.

Several factors can interfere with the quality of a PCR analysis, and the perfect nucleotide identity match in primers/probes could be a determinant for successful amplification^9^. Our results reveal a total hybridization of PCR assays NIID_2019-nCOV_N, nCoV_IP4, and CN-CDC-E with all aligned sequences. In contrast, the assay devised by the US CDC, 2019-nCoV N3 as well as the German Corman-N and the Japanese N_Sarbeco assays, for N gene hybridization, presented mismatches at a 5 bp distance of their 3’ end (Table 1). Base pairing errors in this region can be extremely harmful and directly affect amplification by increasing, on average, by 5 the cycle threshold (Ct) of a PCR analysis, which is a risk factor for a false-negative result^10^ ^,^ ^11^ . 

The assays 2019-nCoV (N1, N2, and N3), NIH-TH_N, nCoV_IP2, CN-CDC-ORF1ab, Charité-E, and E_Sarbeco, presented mismatches located in the 5' or central portion of their primers when aligned with the Brazilian viral genomes (Table 1). Although little information is known regarding the influence that mismatches in these regions of the primers may cause, it is important not to underestimate its potential impact in diagnosis^9^. In addition, mismatches were found for the American and Thai assays for the N gene and the Chinese assay for ORF1ab, in the 5', 3', and central portions and in the 5' portion of the probes, respectively, which might induce a decrease or even an absence of fluorescent signal, promoting unfaithful results^11^. Despite the lack of results on Brazilian sequences, Toms et al.^12^ observed the presence of mismatches in the targets of these assays in several other countries.

**TABLE 1:** List of analyzed assays by targets, frequency and location of mismatches. Each assay below includes three components, 2 primers and 1 probe. Both can be susceptible to matching errors.

Assays/Origen	Target	Total frequency of mismatches	Mismatches at 3' or 5' portion
US-CDC-N1/US-CDC	N	3/177	5’ and 3’
US-CDC-N2/US-CDC	N	2/177	5’
US-CDC-N3/US-CDC	N	3/177	5’ and 3’
NIID_2019-nCOV_N/Japan	N	0/177	-
N_Sarbeco/Japan	N	1/177	3’
CN-CDC-N/China	N	151/177	5’
HKU-N/Hong Kong	N	103/177	5’ and 3’
NIH-TH_N/Thailand	N	2/177	5’
Corman-N/Germany	N	1/177	3’
nCoV_IP2/France	ORF1ab	1/177	5’
nCoV_IP4/France	ORF1ab	0/177	-
CN-CDC-ORF1ab/China	ORF1ab	2/177	5’
Charité-E/Germany	E	2/177	5’
CN-CDC-E/Germany	E	0/177	-
E_Sarbeco/France	E	2/177	5’

Note that the Chinese and Hong Kong assays for the N gene have many mismatches compared to the others. ORF1ab and E targets are less frequent in 3 'mismatches.

Our results demonstrated a higher occurrence of four mismatches from the Chinese CN-CDC-N and HKU-N assays in the Brazilian genomes of SARS-CoV-2. The high frequency (Figure 2A and Figure 2B) of G28881A, G28882A, G28883C (151/177), and T29148C (101/177) mutations inside the target regions of both assays might deeply reduce the accuracy of its use in Brazilian samples. In a previous alignment analysis of 17,175 sequences (including 90 Brazilian sequences), the CN-CDC-N assay presented mismatches in more than 18.8% of the total aligned genomes^1^. In addition, the combination of multiple mismatches, as we found for CN-CDC-N, could directly decrease the amplification performance^9^. A South American study, which included 95 viral sequences deposited by Brazil, also through the GISAID platform, phylogenetically analyzed the genetic diversity of the virus and identified possible mutation hot spots for viral detection within the N gene^13^. This suggests that the use of tests designed by the Chinese CDC and the University of Hong Kong needs to be carefully evaluated, in view of the existence of mutated strains in the targets of these assays in the Brazilian territory. This conclusion was also pointed out by Candido et al.^14^. However, our results include additional data for May, June, and July and take into account only high-coverage sequences. The sequencing quality depends directly on a robust coverage^15^, which is a key factor to be considered, especially in the evaluation of how mismatches could impact diagnosis. 

Interestingly, molecular diagnoses by RT-PCR have shown a conflict with the hospital scenario and clinical parameters. Di Paolo et al.^16^ reported what happened in an intensive care unit in Rome. At the admission of 69 patients on May 19, 13 of them (23.2%) had a high suspicion of presenting the disease based on clinical parameters and chest high-resolution computed tomography (HRCT). However, these patients obtained negative results for diagnostic RT-PCR in three independent analyses. In Beijing, cases of false-negatives by molecular results were identified in two out of ten cases (20%) at the beginning of the pandemic. Considering this issue, the National Health Commission of China established the concept of "Clinical diagnosis" for cases like these, using RT-PCR data for patient isolation matters^17^.

Out of the 177 selected SARS-CoV-2 genome sequences, 77.4% (137/177) were from only two states (Figure 2A), SP and RJ, both located in the southeast region of the country. The 40 remaining sequences were divided among the states of Minas Gerais, Amapá, Pará, Bahia, Alagoas, Acre, Maranhão, Santa Catarina, Amazonas, Espírito Santo and Distrito Federal. SP and RJ were indeed very affected by the pandemic. However, other states, such as Ceará, Bahia, and Pará, were also deeply affected^3^. Nevertheless, the number of complete high-coverage sequences deposited for those states are not representative, or in some cases nonexistent. This vast distinction regarding the number of available sequences between SP and RJ and the other states may be due to their higher Human Development Index (HDI) associated with being the two largest economic centers in Brazil^18^.

Other studies have evaluated the specificity of several PCR assays to sequences from different countries or continents^1^ ^,^ ^12^. They also found that the assays targeting N exhibited more mismatches than the others, which could be due to the existence of more published assays targeting this region. Additionally, the Chinese PCR assays exhibited a higher mismatch than any other assay when aligned to sequences from several countries. Although the mutation rate of SARS-CoV-2 is apparently lower than that of the first SARS-CoV, which has two mutations per human passage^19^, periodic monitoring of the PCR assay protocols is necessary nonetheless.

From the 15 primers and probes analyzed in this study, our results suggest the PCR assays NIID_2019-nCOV_N, nCoV_IP4, and CN-CDC-E are ideal for SARS-CoV-2 diagnosis in Brazil. The selection of assays used in Brazil must be done with caution, considering that the use of primers and probes containing mismatches may lead to false negative results. Certainly, the imprecision of a final result may result from several factors such as incorrect sample handling, transport, storage, insufficient viral material, and contamination^20^, but also from non-functional or poorly validated RT-PCR assays^13^ ^,^ ^16^. Until now, Brazil has not presented studies reporting the rate of false-negative cases of RT-PCR results, but these possible diagnostic failures may lead to incorrect conduct, contribute to breaks in social isolation, and impact on epidemiological surveillance, increasing the risk of infection. 

As the number of high-coverage genomes deposited from states other than SP and RJ was low, our results have a regional bias. It is also important to highlight that we did not perform *in vitro* comparisons between the assays, and our conclusions are based solely on *in silico* analysis.
