# Supplementary material for: In silico analysis of mismatches in RT-qPCR assays of 177 SARS-CoV-2 sequences from Brazil
Source: Rev Soc Bras Med Trop. 2020 Nov 25;53:e20200657. doi: 10.1590/0037-8682-0657-2020 (PMC7723368; doi:10.1590/0037-8682-0657-2020)
Supplement: Supplementary file 1 [file 1678-9849-rsbmt-53-e20200657-suppl1.pdf]

SUPPLEMENTARY DATA S1: Sequences used for alignment, downloaded from the GISAID-EpiCoV platform.

Identification of 177 deposited sequences using Virus name and Accession ID provided by the GISAID-EpiCoV platform.

Virus name	Accession ID
hCoV-19/Brazil/SP-01/2020	EPI_ISL_412964
hCoV-19/Brazil/SP-02/2020	EPI_ISL_413016
hCoV-19/Brazil/SP-03/2020	EPI_ISL_414014
hCoV-19/Brazil/SP-06/2020	EPI_ISL_414015
hCoV-19/Brazil/SP-05/2020	EPI_ISL_414016
hCoV-19/Brazil/SP-04/2020	EPI_ISL_414017
hCoV-19/Brazil/RJ-314/2020	EPI_ISL_414045
hCoV-19/Brazil/BA-312/2020	EPI_ISL_415105
hCoV-19/Brazil/ES-225/2020	EPI_ISL_415128
hCoV-19/Brazil/SP-07/2020	EPI_ISL_416028
hCoV-19/Brazil/SP-08/2020	EPI_ISL_416029
hCoV-19/Brazil/SP-09/2020	EPI_ISL_416031
hCoV-19/Brazil/SP-10/2020	EPI_ISL_416032
hCoV-19/Brazil/SP-11/2020	EPI_ISL_416033
hCoV-19/Brazil/SP-12/2020	EPI_ISL_416034
hCoV-19/Brazil/SP-13/2020	EPI_ISL_416035
hCoV-19/Brazil/SP-14/2020	EPI_ISL_416036
hCoV-19/Brazil/AMBR-02/2020	EPI_ISL_417034
hCoV-19/Brazil/DFBR-0001/2020	EPI_ISL_426580
hCoV-19/Brazil/AL-837/2020	EPI_ISL_427292
hCoV-19/Brazil/BA-510/2020	EPI_ISL_427293
hCoV-19/Brazil/DF-615i/2020	EPI_ISL_427294
hCoV-19/Brazil/DF-619i/2020	EPI_ISL_427295
hCoV-19/Brazil/DF-861/2020	EPI_ISL_427296
hCoV-19/Brazil/DF-862/2020	EPI_ISL_427297
hCoV-19/Brazil/DF-891/2020	EPI_ISL_427298
hCoV-19/Brazil/RJ-352/2020	EPI_ISL_427299
hCoV-19/Brazil/RJ-477/2020	EPI_ISL_427300
hCoV-19/Brazil/RJ-477i/2020	EPI_ISL_427301
hCoV-19/Brazil/RJ-763/2020	EPI_ISL_427302
hCoV-19/Brazil/RJ-818/2020	EPI_ISL_427303
hCoV-19/Brazil/RJ-872/2020	EPI_ISL_427304
hCoV-19/Brazil/SC-766/2020	EPI_ISL_427305
hCoV-19/Brazil/SC-769/2020	EPI_ISL_427306
hCoV-19/Brazil/CV4/2020	EPI_ISL_429667
hCoV-19/Brazil/CV6/2020	EPI_ISL_429669
hCoV-19/Brazil/CV8/2020	EPI_ISL_429671
hCoV-19/Brazil/CV12/2020	EPI_ISL_429674
hCoV-19/Brazil/CV16/2020	EPI_ISL_429676
hCoV-19/Brazil/CV19/2020	EPI_ISL_429679
hCoV-19/Brazil/CV21/2020	EPI_ISL_429681
hCoV-19/Brazil/CV26/2020	EPI_ISL_429684
hCoV-19/Brazil/CV31/2020	EPI_ISL_429687
hCoV-19/Brazil/CV32/2020	EPI_ISL_429688
hCoV-19/Brazil/CV33/2020	EPI_ISL_429689
hCoV-19/Brazil/CV42/2020	EPI_ISL_429695
hCoV-19/Brazil/CV49/2020	EPI_ISL_429702
hCoV-19/Brazil/SP02cc/2020	EPI_ISL_450506
hCoV-19/Brazil/AP161167-IEC/2020	EPI_ISL_450873
hCoV-19/Brazil/PA161548-IEC/2020	EPI_ISL_450874

hCoV-19/Brazil/RJ-899/2020 EPI_ISL_456071
hCoV-19/Brazil/RJ-1056/2020 EPI_ISL_456072
hCoV-19/Brazil/RJ-1058/2020 EPI_ISL_456073
hCoV-19/Brazil/RJ-1065/2020 EPI_ISL_456074
hCoV-19/Brazil/RJ-1100/2020 EPI_ISL_456075
hCoV-19/Brazil/RJ-1111/2020 EPI_ISL_456076
hCoV-19/Brazil/RJ-1119/2020 EPI_ISL_456077
hCoV-19/Brazil/RJ-1402/2020 EPI_ISL_456079
hCoV-19/Brazil/RJ-1464/2020 EPI_ISL_456080
hCoV-19/Brazil/RJ-1466/2020 EPI_ISL_456081
hCoV-19/Brazil/RJ-1600/2020 EPI_ISL_456082
hCoV-19/Brazil/RJ-1627/2020 EPI_ISL_456083
hCoV-19/Brazil/RJ-1690/2020 EPI_ISL_456084
hCoV-19/Brazil/RJ-1691/2020 EPI_ISL_456085
hCoV-19/Brazil/RJ-1701/2020 EPI_ISL_456086
hCoV-19/Brazil/RJ-1702/2020 EPI_ISL_456087
hCoV-19/Brazil/RJ-1719/2020 EPI_ISL_456088
hCoV-19/Brazil/RJ-1901/2020 EPI_ISL_456089
hCoV-19/Brazil/RJ-1902/2020 EPI_ISL_456090
hCoV-19/Brazil/RJ-1921/2020 EPI_ISL_456091
hCoV-19/Brazil/RJ-1923/2020 EPI_ISL_456092
hCoV-19/Brazil/RJ-1927/2020 EPI_ISL_456093
hCoV-19/Brazil/RJ-1943/2020 EPI_ISL_456094
hCoV-19/Brazil/RJ-1948/2020 EPI_ISL_456095
hCoV-19/Brazil/RJ-1952/2020 EPI_ISL_456096
hCoV-19/Brazil/RJ-1966/2020 EPI_ISL_456097
hCoV-19/Brazil/RJ-2000/2020 EPI_ISL_456098
hCoV-19/Brazil/RJ-2007/2020 EPI_ISL_456099
hCoV-19/Brazil/RJ-2033/2020 EPI_ISL_456100
hCoV-19/Brazil/RJ-2044/2020 EPI_ISL_456101
hCoV-19/Brazil/RJ-2057/2020 EPI_ISL_456102
hCoV-19/Brazil/RJ-2062/2020 EPI_ISL_456103
hCoV-19/Brazil/RJ-2072/2020 EPI_ISL_456104
hCoV-19/Brazil/RJ-2077/2020 EPI_ISL_456105
hCoV-19/Brazil/RJ-2078/2020 EPI_ISL_456106
hCoV-19/Brazil/AP162741-IEC/2020 EPI_ISL_458138
hCoV-19/Brazil/AC162535-IEC/2020 EPI_ISL_458139
hCoV-19/Brazil/PA162802-IEC/2020 EPI_ISL_458140
hCoV-19/Brazil/PA164239-IEC/2020 EPI_ISL_458141
hCoV-19/Brazil/AP162966-IEC/2020 EPI_ISL_458142
hCoV-19/Brazil/AP164082-IEC/2020 EPI_ISL_458143
hCoV-19/Brazil/AP163972-IEC/2020 EPI_ISL_458144
hCoV-19/Brazil/AP164346-IEC/2020 EPI_ISL_458145
hCoV-19/Brazil/PA164173-IEC/2020 EPI_ISL_458146
hCoV-19/Brazil/PA164218-IEC/2020 EPI_ISL_458147
hCoV-19/Brazil/PA164684-IEC/2020 EPI_ISL_458148
hCoV-19/Brazil/MA163069-IEC/2020 EPI_ISL_458149
hCoV-19/Brazil/RJ-1555/2020 EPI_ISL_467344
hCoV-19/Brazil/RJ-1574/2020 EPI_ISL_467345
hCoV-19/Brazil/RJ-1595/2020 EPI_ISL_467346
hCoV-19/Brazil/RJ-2091/2020 EPI_ISL_467347
hCoV-19/Brazil/RJ-2195/2020 EPI_ISL_467348
hCoV-19/Brazil/RJ-2197/2020 EPI_ISL_467349
hCoV-19/Brazil/RJ-2208/2020 EPI_ISL_467350

hCoV-19/Brazil/RJ-2233/2020	EPI_ISL_467351
hCoV-19/Brazil/RJ-2422/2020	EPI_ISL_467352
hCoV-19/Brazil/RJ-2669/2020	EPI_ISL_467353
hCoV-19/Brazil/RJ-2676/2020	EPI_ISL_467354
hCoV-19/Brazil/RJ-2678/2020	EPI_ISL_467355
hCoV-19/Brazil/RJ-2682/2020	EPI_ISL_467356
hCoV-19/Brazil/RJ-2683/2020	EPI_ISL_467357
hCoV-19/Brazil/RJ-2696/2020	EPI_ISL_467358
hCoV-19/Brazil/RJ-2717/2020	EPI_ISL_467359
hCoV-19/Brazil/RJ-2733/2020	EPI_ISL_467360
hCoV-19/Brazil/RJ-2769/2020	EPI_ISL_467361
hCoV-19/Brazil/RJ-2770/2020	EPI_ISL_467362
hCoV-19/Brazil/RJ-2776/2020	EPI_ISL_467363
hCoV-19/Brazil/RJ-2777/2020	EPI_ISL_467364
hCoV-19/Brazil/RJ-2811/2020	EPI_ISL_467365
hCoV-19/Brazil/RJ-2812/2020	EPI_ISL_467366
hCoV-19/Brazil/RJ-2822/2020	EPI_ISL_467367
hCoV-19/Brazil/RJ-2840/2020	EPI_ISL_467368
hCoV-19/Brazil/RJ-2844/2020	EPI_ISL_467369
hCoV-19/Brazil/RJ-2847/2020	EPI_ISL_467370
hCoV-19/Brazil/RJ-2868/2020	EPI_ISL_467371
hCoV-19/Brazil/SP-138/2020	EPI_ISL_468305
hCoV-19/Brazil/SP-139/2020	EPI_ISL_468306
hCoV-19/Brazil/SP-140/2020	EPI_ISL_468307
hCoV-19/Brazil/SP-141/2020	EPI_ISL_468308
hCoV-19/Brazil/SP-144/2020	EPI_ISL_468310
hCoV-19/Brazil/SP-146/2020	EPI_ISL_468311
hCoV-19/Brazil/SP-147/2020	EPI_ISL_468312
hCoV-19/Brazil/SP-148/2020	EPI_ISL_468313
hCoV-19/Brazil/SP-149/2020	EPI_ISL_468314
hCoV-19/Brazil/SP-500/2020	EPI_ISL_468315
hCoV-19/Brazil/SP-504/2020	EPI_ISL_468316
hCoV-19/Brazil/SP-505/2020	EPI_ISL_468318
hCoV-19/Brazil/SP-506/2020	EPI_ISL_468319
hCoV-19/Brazil/SP-508/2020	EPI_ISL_468320
hCoV-19/Brazil/SP-516/2020	EPI_ISL_468321
hCoV-19/Brazil/SP-47/2020	EPI_ISL_471539
hCoV-19/Brazil/SP-126/2020	EPI_ISL_471541
hCoV-19/Brazil/SP-127/2020	EPI_ISL_471542
hCoV-19/Brazil/SP-131/2020	EPI_ISL_471543
hCoV-19/Brazil/SP-601/2020	EPI_ISL_471545
hCoV-19/Brazil/SP-537/2020	EPI_ISL_471546
hCoV-19/Brazil/SP-606/2020	EPI_ISL_471548
hCoV-19/Brazil/SP-607/2020	EPI_ISL_471549
hCoV-19/Brazil/SP-545/2020	EPI_ISL_471551
hCoV-19/Brazil/SP-549/2020	EPI_ISL_471552
hCoV-19/Brazil/SP-551/2020	EPI_ISL_471554
hCoV-19/Brazil/SP-512/2020	EPI_ISL_471556
hCoV-19/Brazil/SP-523/2020	EPI_ISL_471562
hCoV-19/Brazil/SP-524/2020	EPI_ISL_471581
hCoV-19/Brazil/SP-525/2020	EPI_ISL_471582
hCoV-19/Brazil/SP-526/2020	EPI_ISL_471647
hCoV-19/Brazil/SP-527/2020	EPI_ISL_471648
hCoV-19/Brazil/RJ01/2020	EPI_ISL_483065

Rev Soc Bras Med Trop | on line | Vol.:53:(e2020657): 2020

hCoV-19/Brazil/HIAE-SP03/2020 EPI_ISL_486427
hCoV-19/Brazil/HIAE-SP04/2020 EPI_ISL_486429
hCoV-19/Brazil/UFRJ-IBEX_1019/2020 EPI_ISL_492032
hCoV-19/Brazil/UFRJ-IBEX_1640/2020 EPI_ISL_492033
hCoV-19/Brazil/UFRJ-IBEX_2215/2020 EPI_ISL_492034
hCoV-19/Brazil/UFRJ-IBEX_2517/2020 EPI_ISL_492035
hCoV-19/Brazil/UFRJ-IBEX_9331/2020 EPI_ISL_492036
hCoV-19/Brazil/UFRJ-IBEX_11784/2020 EPI_ISL_492037
hCoV-19/Brazil/UFRJ-IBEX_11785/2020 EPI_ISL_492038
hCoV-19/Brazil/UFRJ-IBEX_11786/2020 EPI_ISL_492039
hCoV-19/Brazil/UFRJ-IBEX_32317/2020 EPI_ISL_492040
hCoV-19/Brazil/UFRJ-IBEX_32321/2020 EPI_ISL_492041
hCoV-19/Brazil/UFRJ-IBEX_54093/2020 EPI_ISL_492042
hCoV-19/Brazil/UFRJ-IBEX_57437/2020 EPI_ISL_492043
hCoV-19/Brazil/UFRJ-IBEX_57721/2020 EPI_ISL_492044
hCoV-19/Brazil/UFRJ-IBEX_57722/2020 EPI_ISL_492045
hCoV-19/Brazil/UFRJ-IBEX_58090/2020 EPI_ISL_492046
hCoV-19/Brazil/UFRJ-IBEX_58110/2020 EPI_ISL_492047
hCoV-19/Brazil/UFRJ-IBEX_58271/2020 EPI_ISL_492048
